# Prevalence, risk factors and molecular characterization of *Chlamydia trachomatis* in pregnant women from Córdoba, Argentina: A prospective study

**DOI:** 10.1371/journal.pone.0217245

**Published:** 2019-05-31

**Authors:** Ana Ximena Kiguen, Marcela Marramá, Susana Ruiz, Patricia Estofan, Raúl Fernando Venezuela, Jessica Paola Mosmann, Marina Soledad Monetti, Virginia Rivero, Cecilia Gabriela Cuffini

**Affiliations:** 1 Instituto de Virología Doctor José María Vanella, Facultad de Ciencias Médicas, Universidad Nacional de Córdoba, Córdoba, Argentina; 2 Dirección de Especialidades Médicas, Municipalidad de Córdoba, Córdoba, Argentina; 3 Laboratorios LACE Sociedad Anónima, Córdoba, Argentina; 4 Centro Integral de Ginecología, Obstetricia y Reproducción (CIGOR), Córdoba, Argentina; 5 Centro de Investigaciones en Bioquímica Clínica e Inmunología (CIBICI-CONICET), Departamento de Bioquímica Clínica, Facultad de Ciencias Químicas, Universidad Nacional de Córdoba, Córdoba, Argentina; University of Texas Health Science Center at San Antonio, UNITED STATES

## Abstract

**Background:**

*Chlamydia trachomatis* causes the most prevalent bacterial Sexual Transmitted Infection. In pregnant women, untreated chlamydial infections are associated with abortions, premature rupture of membranes, postpartum endometritis, low birth weight and transmission to the newborn. In Córdoba, Argentina, there is little knowledge about the prevalence of *Chlamydia trachomatis* in women in their third trimester of pregnancy, so, the aim of this study was to evaluate *Chlamydia trachomatis* prevalence and genotypes present in Cordovan pregnant women with different age and socioeconomic status.

**Methods and findings:**

Design: prospective study.

Settings: Women population from Cordoba city, Argentina.

Population: Pregnant women having 35 to 37 weeks of gestation.

Methods: Five hundred and nine cervical swabs were collected. Each sample was subjected to DNA extraction and PCR for *Chlamydia trachomatis* using primers NRO/NLO and CTP1/CTP2. Positives samples were sequenced to determine genotype. Main outcome measures: Demographic data of the patients were collected to detect a population at risk for this infection.

**Results:**

A prevalence of 6.9% (35/509) for *Chlamydia trachomatis* infection was detected, with 32/295 and 3/214 from pregnant women with low or better economic resources respectively (p = 0,0001). Results showed a significantly increased rate of 11.6% (30/258) in women under 25 years compared with 2% (5/251) in patients over that age (p = 0,00003). Genotype E was the most prevalent.

**Conclusions:**

With these results, we can say that pregnant women under 25 years old and low economic resources are one of the populations in which the screening programs of *Chlamydia trachomatis* should focus.

## Introduction

*Chlamydia trachomatis* (*C*. *trachomatis*) is an obligate intracellular Gram negative bacterium causing the most prevalent bacterial Sexual Transmitted Infection (STI) throughout the world. The World Health Organization (WHO) estimated that annually some 357 million people get one STI include: chlamydia, gonorrhea, syphilis or trichomoniasis [1]. According to ompA gene molecular differences, *C*. *tracho*matis could be divided into different genotypes that are responsible for various diseases: genotypes A, B, Ba and C causing trachoma; genotypes D, Da, E, F, G, H, I, Ia, J and K responsible for urogenital infections in adults and respiratory and conjunctival infections in neonates and genotypes L1, L2, L2a and L3 causing Lymphogranuloma venereum [2, 3]. The most important characteristic of *C*. *trachomatis* is the ability to produce acute complications and long-term sequelae in upper genital tract, thus affecting the reproductive health.

In women, *C*. *trachomatis* infection presents asymptomatically in 70–75% of cases. Eng and Butler found that 30–40% of sexually active teenagers were infected [4] and up to 40% of them may develop pelvic inflammatory disease (PID) if not received the specific antimicrobial treatment [5]. *C*. *trachomatis* mainly affects women and adolescents younger than 20 years old. It has been postulated the immature cervix is more susceptible to *C*. *trachomatis* infection, so younger women are more prone to infection than older women [6, 7].

Infection prevalence reported in pregnant women is 3.5% in USA [8], 12.1% in the UK [9], 6.4% in Australia [10], 11% in Brazil and 10% in Perú [11, 12]. Untreated chlamydial infection in pregnant women is associated with miscarriage, postpartum endometritis, premature rupture of membranes, low birth weight and transmission to the newborn [13]. Some studies suggest that the risk of infection in a newborn from infected mothers is about 50% and can cause bronchitis, pneumonia and neonatal conjunctivitis [14].

The antimicrobial treatment for pregnant patients with *C*. *trachomatis* infection is Erythromycin or Azithromycin. The 1980s implementation of antenatal screening and treatment for chlamydial infection in the USA significantly lowered the incidence of both neonatal chlamydial pneumonia and conjunctivitis, which was previously the most common cause of neonatal conjunctivitis there [15]

Currently, there are no programs routinely conducted for *C*. *trachomatis* screening in antenatal care in Latin America, and there are no WHO recommendations for *C*. *trachomatis* screening and treatment in pregnant women [16]. In addition, in Córdoba, Argentina, there is little knowledge about the prevalence of *C*. *trachomatis* in women in their third trimester of pregnancy. Taking into account data mentioned above, in this work we evaluated *C*. *trachomatis* prevalence and genotypes present in Cordovan pregnant women with different age and socioeconomic status. Our results demonstrated that younger and low income pregnant women are most affected.

## Methods

### Study design

We conducted a prospective study with patients from two Health Centers in Córdoba, Argentina: Medical Specialties of Córdoba Municipality (MSCM) and a private laboratory from Cordoba city named LACE (LACE). Both MSCM participants (n = 301) and LACE participants (n = 208) were recruited between September 2014 and February 2015. Women ≥ 14 years old having 35 weeks of gestation and interested in participate were enrolled after providing informed consent approved by the Ethics Committee (C.I.E.S. Oulton- Romagosa. Date of approval: 03/12/2014. Reference number: 014). It is important to emphasize that the Ethics Committee approved the lack of parent or guardian consent in patients under 18 years, according to the law 26,742 (Rights of the Patient in its relationship with Professionals and Health Institutions). Health care professionals collected demographic data and swabs samples from each patient. It should be noted that none of the patients enrolled in this study had compatible symptoms with *C*. *trachomatis* infection.

Clinical Samples: Five hundred and nine cervical swabs were collected from pregnant women. All samples were placed in sterile tubes containing 1 ml of SPG (sucrose, phosphate, glutamic acid), and subsequently sent to the Instituto de Virología, Facultad de Ciencias Médicas, Universidad Nacional de Córdoba, Argentina to be processed.

DNA Extraction: Two hundred μl of each sample were subjected to DNA extraction using the Accuprep Genomic DNA Extraction Kit (BIONEER, Alameda, CA, USA) according to the manufacturer’s instructions.

OmpA gene PCR: PCR DNA extract (5 μl) was used to amplify a 1087 pb fragment of the ompA gene of *C*. *trachomatis*, using primers NRO (5’CTCAACTGTAACTGCGTATTT3’) and NLO (5’ATGAAAAAACTCTTGAAATCG3´). PCR amplification processes commenced with a 4-minute denaturation step at 95°C and continued with 49 amplification cycles. Each cycle consisted of a first denaturation step at 95°C for 1 min, an annealing step at 55°C for 1 min and a final step of chain elongation at 72° C for 1.5 min [17].

Cryptic Plasmid PCR: The primers used to generate a 201-bp fragment from the cryptic plasmid of *C*. *trachomatis* were CTP1 (5'-TAGTAACTGCCAClTCATCA-3') and CTP2 (5'-TTCCCCTTGTAATTCGTTGC-3'). The PCR amplification consisted of DNA denaturation at 95°C for 4 min followed by 35 cycles of amplification. Each cycle consisted of 1 min at 95°C, 1 min at 55°C and 1.5 min at 72°C followed by a final elongation at 72°C for 4 min. The ompA gene and cryptic plasmid PCR products were visualized after electrophoresis in a 1% agarose gel by ECO-Gel 20.000X Highway staining [17]. Positive and negative controls were used in all determinations of PCR (dx.doi.org/10.17504/protocols.io.zeef3be).

Sequencing of the ompA gene: For sequence analysis, the PCR products were purified with the QIAquick Gel Extraction Kit (Qiagen, Valencia, CA, US) and subjected to direct nucleotide sequencing reaction in both directions using an ABI automatic sequencer. The sequences were analyzed using the Molecular Evolutionary Genetics Analysis software package, MEGA 6 [18]. Sequences of the ompA derived from strains used in this study were analyzed along with the next sequences from strains available in GenBank: VR-348-E (accession number JX559522.1), VR-346-F (JX564244.1), VR-885-D (JX559520.1), VR-1477-C (559519.1), VR-573-B (JX559518.1), VR-347-Ba (KP120856.1), VR-878-G (JX564245.1), VR-879-H (JX564246.1), VR-880-I (JX564247.1), VR-886-J(JX648604.1), VR-887-K (JX564248.1), VR-901B-L1 (JX569832.1), VR-577-L2 (JX569836.1), VR-903-L3 (JX569834.1), China-F (EU339316), Tailandia-F (KM369937), Rusia-F (KU963178), Holanda-F (AF265240), Australia-F (AY464145), Brasil-F (DQ442881), USA-F (CP006674), Dinamarca-F (AM901152), Japon-F (AB915586), Argentina-D (EU191085), India-D (KP015822), USA-D (CP00677), Francia-D (X62919), Japon-D (AB915583), Dinamarca-D(AM901213), Australia-D (AY464176), Brasil-D (DQ442877), Islandia-D (AF414950), Suiza-L2 (DQ217607), Portugal-L2 (EU296834), Holanda-L2 (AY586530), USA-L2 (CP002682), Japon-L2 (AB915593), Japon-E (AB915585), Rusia-E (KU963184), Argentina-E (KC120818), Dinamarca-E (AM901208), Tailandia-E (KM369935), Australia-E (AY464144), Francia-E (JN192145), India-E (KP015823), Grecia-E (HQ637270), Brasil-E (FJ418802), Argentina-E (DQ890028), Holanda-E (AF265237), USA-E (CP006675), USA-B (DQ064297), Italia-B (U80075), Australia-B (AY464143), UK-B (M33636), Argentina-G (KC120822), Argentina-G (KC120825), India-G (KP015825), Australia-G (AY464159), USA-G (DQ064299), Japon-G (AB915587), Dinamarca-G (AM901157), India-Ia (KP015824), Japon-Ia (AB915589), USA-Ia (AF063201), Portugal-Ia (DQ116398), USA-Ia (DQ064291), Argentina-Ia (EU000492), and the tree was rooted with the ompA sequence of the *Chlamydia suis* (*C*. *suis*) strain (accession number AY687631). Phylogenetic tree was constructed using the neighbor-joining method. Branching pattern confidence levels were estimated by the bootstrap resampling of the data based on 1000 random replicates.

Statistical Analysis: A descriptive analysis of the sociodemographic characteristics of the patients was carried out. The results of the diagnostic tests were also analyzed. The normal distribution of the variables were expressed by the average and standard deviation and categorized. The prevalence of *C*. *trachomatis* infection was calculated with their respective 95% CI. Possible risk factors were evaluated using bivariate and multivariate logistic regression analysis. Values of p <0.05 were considered significant. The analysis was performed with Epi info software [19].

## Results

We studied patients from 14 to 43 years of age with a median of 25 years. From 509 samples analyzed in the present work, 35 were positive for *C*. *trachomatis* DNA, revealing a total prevalence of 6.9% in the pregnant population included in our study.

Thirty two samples were from patients with Low Economic Resources (LER) while the 3 remaining had Better Economic Resources (BER), revealing a very increased positivity in the first patients mentioned (p 0,0001). In order to establish the LER, the patients had to be under a Program called Universal Assignment per child. It is an economic aid provided by the Argentine State to people with very low resources. Prevalence studies showed that positive samples belong mainly to young women. The positive samples were distributed in different age groups as can be seen in Table 1. Combining the age groups, we observed a significantly increased rate of 11.6% (30/258) in women under 25 years, compared with 2% (5/251) in patients over that age (p = 0,00003). It is interesting to note that pregnant patients between 21 and 25 years old have 2,9 more risk of suffering *C*. *trachomatis* infection than the rest of them (OR: 2,9. CI: 1,4–5,8. p = 0,001) (Table 1).

**Table 1:** Demographic distribution.

	*C*. *trachomatis* + n (%)	*C*. *trachomatis*- n (%)	OR (CI)	p	Total n (%)
**Age Range**					
< 16	2 (0,4)	6 (1,2)	4,7 (0,9–24,3)	0,04	**8** **(1,6)**
16–20	10 (2)	96 (18,9)	1,5 (0,7–3,3)	0,24	**106 (20,8)**
21–25	18 (3,5)	126 (24,7)	2,9 (1,4–5,8)	0,001	**144 (28,3)**
26–30	3 (0,6)	105 (20,6)	0,3 (0,09–1)	0,05	**108 (21,2)**
31–35	1 (0,2)	96 (18,9)	0,1 (0,01–0,8)	0,01	**97** **(19)**
36–40	1 (0,2)	39 (7,6)	0,3 (0,04–2,4)	0,2	**40** **(7,9)**
>40	0 (0)	6 (1,2)			**6** **(1,2)**
**Economic Level**					
LER	32 (6,3)	262 (51,5)	8,6 (2,6–28,5)	0,00002	**294** **(57,8)**
BER	3 (0,6)	212 (41,6)	0,1 (0,03–0,3)	0,00002	**215** **(42,2)**

The frequencies, percentages OR (IC) and p of the demographic characteristics of all pregnant women admitted to the study are shown.

**Fig 1:**
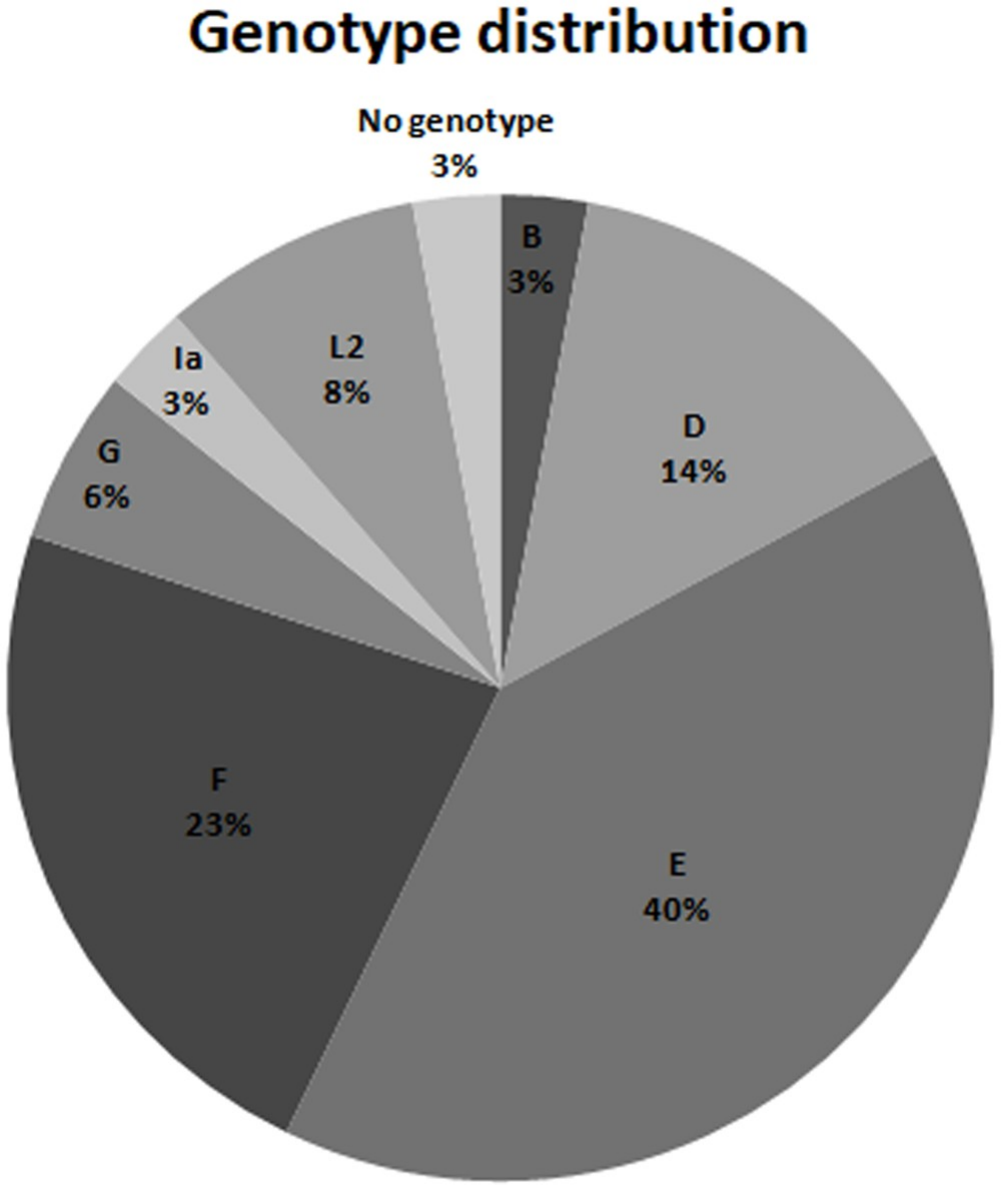
Genotype distribution. The distribution of *C*. *trachomatis* genotypes found in pregnant women is shown.

Using OmpA sequencing *C*. *trachomatis* samples showed 7 different genotypes (B, D, E, F, G, Ia and L2, Fig 1) of which genotype E was the most prevalent, as can be seen in Fig 1.

**Fig 2:**
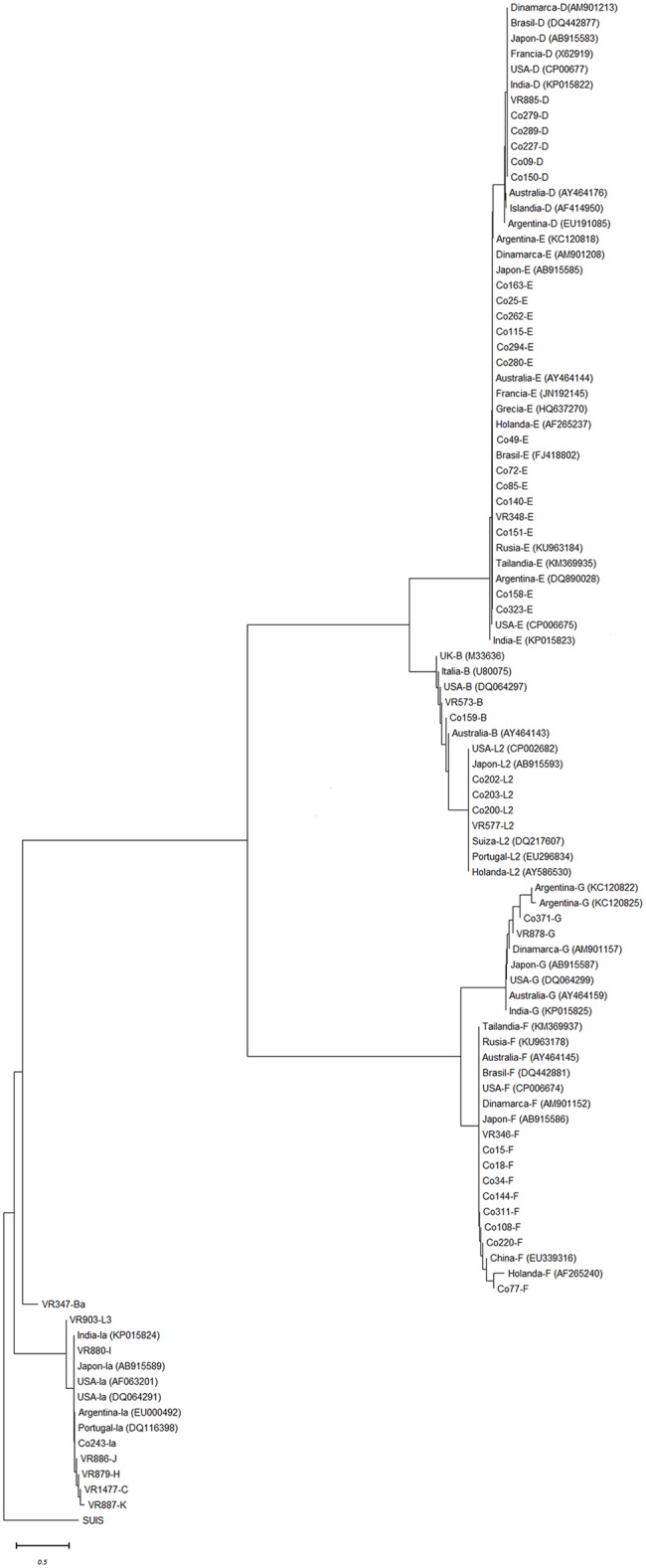
Phylogenetic tree of the *OmpA* gene of *C*. *trachomatis*. The neighbor-joining method was used in MEGA& to generate phylogenetic trees for ompA from DNA sequences (596bp). Initial nucleotide alignments generated with strains that belong to this study are initiated by CO.

When a phylogenetic analysis was performed in sequenced samples three constructed with three major subdivisions could be detected. Main branches do not match the patterns of tissue tropisms and associated clinical presentation *C*. *trachomatis* infection in human hosts. (Fig 2).

## Discussion

In our knowledge this is the first report of *C*. *trachomatis* prevalence in women in their third trimester of pregnancy from Cordoba, Argentina. According to published studies, the prevalence of this bacterium differs greatly around the world, with values between 0.1% and 25.7% depending on the particular characteristics of the pregnant population and the methodology used in the study [20, 21]. In Latin America, there are a few studies regarding *C*. *trachomatis* prevalence in pregnant women and values reported are 5.9%, 10% and 11% for pregnant women from Chile, Peru and Brazil respectively [22, 12, 23]. In the present study we found a prevalence of 6.9%, which is within the range expected for the region. Highest percentages of positivity were found in women between 21 to 25 years and with limited financial resources. Young women are a vulnerable group of the population with regard to sexually transmitted diseases. This is thought to be because of the relative immaturity of the genital tract, making it prone to both trauma and infection, particularly in the developing transformation zone of the cervix [6–7].

One of the limitations of this study is that there is an inevitable selection bias due to the object of study, since women older than 25 years infected with *C*. *trachomatis*, might not have entered it because the complications of this infection did not allow their pregnancy.

The prevalence of 6.9% detected in pregnant women in our study is lower than that reported in asymptomatic young woman in Cordoba city, in which a prevalence of 8.9% was observed [24], and it is greater than that found by Zucotti et al. in pregnant patients in the first trimester who are seen in a private center where patients must pay for each medical practice they need [25].

This might be due to the fact that the population studied by Zucotti has a good economic level, which is why, according to our results; it is a protective factor for *C*. *trachomatis* infection.

As can be seen in Table 1, patients with LER have 8 times more risk of having *C*. *trachomatis* infection than patients with BER (OR: 8.6 CI: 2.6–28.5); the opposite happens with patients with BER since, according to statistics, this characteristic is protective for the aforementioned infection (OR: 0.1 CI: 0.03–0.3).

There are mechanisms that can explain the association of *C*. *trachomatis* infection with low socioeconomic status; these include a lower commitment to the control activities of STI, for example, not attending the instances of diagnosis of them. In addition, there would be individual, family and community factors that would negatively impact on safe sexual practices, such as the use of condoms [26].

The majority of published data clearly show that genotypes E and D are the most frequently isolated from genital tract infections [27]. Most traditional genotypes in patients with urogenital infection would be E, F, D, G and K, constituting between 60 and 80% of positive patients [28]. In our study genotypes E and F were the most frequently found in sequenced samples from pregnant women, followed by genotypes D, L2, G, B and Ia. Sexually active, asymptomatic patients from Córdoba, Argentina also showed genotype E as the most frequently detected, followed by genotype D and less frequently genotypes F and G [29]. In addition, genotypes E and D were the most frequently isolated in a study conducted in Buenos Aires, Argentina in adults and neonates with ophthalmia neonatarum [30].

In general, our results are consistent with those reported in most published studies, both in Argentina [29, 30] and elsewhere in the world, which detects a higher prevalence of genotype E, D and F in genital infections. However, our study reported also genotype L2, a finding not usually reported in bibliography.

Regarding the phylogenetic analysis, the distribution of genotypes observed in our study was similar to the distribution reported by Brunelle et al [31], which suggest that there is a marked trend in the group of serovars, which is not related to tissue tropism. Similar results were obtained by Monetti et al and Lutter et al [17, 32], who also proposes that the MOMP variability is due to antigenicity there of and immune selective pressure.

*C*. *trachomatis* infections are accompanied by psychosocial and economic complications. For this reason, screening programs have been implemented in different countries. Their main purpose is to reduce morbidity through early detection and appropriate specific treatment. As a secondary objective of these programs is to decrease the overall prevalence of *C*. *trachomatis* and subsequently reduce transmission in the population.

With regard to financial costs, it is considered that a screening program is effective when the costs associated with the logistics of screening and treatment of positive cases is less than or equal to the diagnosis and treatment of complications. Some publications show that screening programs put in place for this infection are profitable in selected populations [33–35].

With the data presented here, we would be able to state that low income pregnant women less than 25 years would be one of the populations selected for *C*. *trachomatis* screening programs.
